# Dietary Cholesterol-Induced Post-Testicular Infertility

**DOI:** 10.1371/journal.pone.0026966

**Published:** 2011-11-02

**Authors:** Aurélia Ouvrier, Georges Alves, Christelle Damon-Soubeyrand, Geoffroy Marceau, Rémi Cadet, Laurent Janny, Florence Brugnon, Ayhan Kocer, Aurélien Pommier, Jean-Marc A. Lobaccaro, Joël R. Drevet, Fabrice Saez

**Affiliations:** 1 Equipe “Epididyme et maturation des gamètes”, Clermont Université, Université Blaise Pascal, UMR GReD (Génétique Reproduction & Développement) CNRS 6247, INSERM U931, Aubière, France; 2 Equipe “LXR, oxystérols et tissus stéroïdogènes” & Centre de Recherche en Nutrition Humaine d'Auvergne (CRNH), Clermont Université, Université Blaise Pascal, UMR GReD (Génétique Reproduction & Développement) CNRS 6247, INSERM U931, Aubière, France; 3 Equipe “Implications métaboliques et moléculaires des rétinoïdes dans le développement humain”, Clermont Université, Université Blaise Pascal, UMR GReD (Génétique Reproduction & Développement) CNRS 6247, INSERM U931, Aubière, France; 4 Université Clermont 1, EA 975, Biologie de la Reproduction, Clermont-Ferrand, France; 5 CHU Estaing, Assistance Médicale à la Procréation, CECOS, Clermont-Ferrand, France; Ecole Normale Supérieure de Lyon, France

## Abstract

This work shows that an overload of dietary cholesterol causes complete infertility in dyslipidemic male mice (the Liver X Receptor-deficient mouse model). Infertility resulted from post-testicular defects affecting the fertilizing potential of spermatozoa. Spermatozoa of cholesterol-fed *lxr−/−* animals were found to be dramatically less viable and motile, and highly susceptible to undergo a premature acrosome reaction. We also provide evidence, that this lipid-induced infertility is associated with the accelerated appearance of a highly regionalized epididymal phenotype in segments 1 and 2 of the caput epididymidis that was otherwise only observed in aged LXR-deficient males. The epididymal epithelial phenotype is characterized by peritubular accumulation of cholesteryl ester lipid droplets in smooth muscle cells lining the epididymal duct, leading to their transdifferentiation into foam cells that eventually migrate through the duct wall, a situation that resembles the inflammatory atherosclerotic process. These findings establish the high level of susceptibility of epididymal sperm maturation to dietary cholesterol overload and could partly explain reproductive failures encountered by young dyslipidemic men as well as ageing males wishing to reproduce.

## Introduction

Cellular and plasma cholesterol levels are tightly controlled to prevent excessive accumulation of cholesterol in tissues [1]. Dyslipidemia is on the rise in young people in both developed and developing countries, with major effects on the incidence of life-threatening conditions such as obesity and associated cardiovascular complications [2]. Perhaps less recognized but growing in importance are effects of lipid disorders on reproductive fitness [3], [4], [5]. Among transcription factors regulating cholesterol homeostasis, Liver X Receptors α (LXRα – Nr1h3) and β (LXRβ – Nr1h2) play central roles in various cell types. Both are activated by metabolic derivatives or oxidized forms of cholesterol, and have been shown to control the expression of a wide spectrum of genes that determine lipid and metabolic homeostasis, energy utilization, differentiation, proliferation, inflammation, and reproduction [6], [7], [8], [9].

Male mice deficient for the two LXR isoforms (LXRα and LXRβ) become subfertile upon ageing and are totally infertile at 8–9 months, showing both a testicular phenotype and a caput epididymidis phenotype restricted to the proximal caput [10], [11], [12]. The caput epididymal tissue defect is characterized by cholesteryl ester (CE) accumulation [12] and a luminal compartment filled with amorphous material [10]. In addition, mature spermatozoa retrieved from the cauda epididymidis of old *lxrα;β-/−* animals show structural fragility at the head/midpiece junction resulting in abundant broken sperm cells [10]. More recently, we reported that *lxr* disruption provokes CE accumulation in a particular cell subtype of the caput epididymidal epithelium, the so-called apical cells, following down-expression of the ATP-binding cassette transporter A1 (ABCA1) [11]. In addition to the epithelial apical cell-located lipid accumulation, peritubular CE accumulation was also observed in the proximal caput epididymidis of *lxrα;β−/−* animals [11]. At 3–4 months of age, LXR-deficient male mice are totally fertile and do not show any phenotype at all [8], [10]. Young LXR-deficient male mice are thus a good model to address the question of how an excess of dietary lipid affects reproductive functions in dyslipidemic animals.

## Experimental Procedures

### Animals

Lxr-knockout mice [7], [13] were maintained on a mixed strain background (C57BL/6:129Sv) and were housed in an animal facility with controlled environment (22°C, 12 hr light/12 hr dark). Under control conditions, mice were fed *ad libitum* a Global-diet_2016S (Harlan, Gannat, France). Under high-cholesterol-diet (HCD), 3-month-old males were fed for 4 weeks a lipid-enriched diet containing 1.25% cholesterol (Safe, Augy, France). Mouse housing and manipulation were approved by the Regional Ethic Committee in Animal Experimentation (authorization CE2-04). For fertility tests, virgin 10-week-old SWISS females were used. Wild-type (*wt*) and *lxrα;β−/−* male mice (hybrid line C57BL6x129 SVJ) [14], were killed by decapitation.

### Fertility

Six *wt* and *lxrα;β−/−* male mice at 4 months of age, fed a HCD or control diet for 4 weeks, were each mated with 2 SWISS *wt* females. The fertility test was made during the last 8 days of the diet, food was removed during the 12 hr of dark (mating period, 1 male with 2 females in each cage). Males and females were separated every day for the 12 hr of light, and HCD was given only to the males. At the end of the 8-day mating period, males were killed and the females housed in individual cages to follow gestations and deliveries.

### Sperm preparation

Epididymides were removed and, after dissection, the cauda epididymidis was transferred to a small glass dish containing 500 µL of Whitten's HEPES medium (WH) (20 mM HEPES, pH 7.3, 100 mM NaCl, 4.7 mM KCl, 1.2 mM KH_2_PO_4_, 1.2 mM MgSO_4_, 5.5 mM glucose, 1 mM pyruvic acid, 4.8 mM lactic acid, Sigma, St Quentin Fallavier, France). To recover sperm cells, repeated punctures with a 26-gauge needle were made. After 5 min of incubation at 37°C to allow for sperm cell dispersion, sperm suspensions were recovered.

### Sperm counts

After dilution of the original cell suspension (1/50 in PBS), spermatozoa were counted in a Malassez hemocytometer.

### Motility

Computer-assisted sperm analysis parameters were determined immediately after collection. Sperm tracks (300 frames) were captured using a CEROS sperm analysis system (Hamilton Thorne, Lisieux, France; software version 12).

### Viability

Twenty µL of sperm suspension and 20 µL of eosin solution (0.01 g.mL^−1^) were mixed, thirty seconds later, 30 µL of nigrosin suspension (0.07 g.mL^−1^) were added and vortexed for 2 sec. Then, 30 µL of the suspension were recovered and spread on a glass slide. After drying at room temperature, sperm viability was determined: dead and living spermatozoa had pink and white colored heads, respectively. A minimum of 200 spermatozoa was counted per sample.

### Acrosomal status

Fifty µL of sperm suspension were diluted in 450 µL of WH medium and were then fixed by slowly adding 500 µL PBS-paraformaldehyde (PFA) 4% w/v and incubated for 30 min at 4°C. Cells were then centrifuged at 500 g for 5 min, washed twice with PBS, finally diluted in PBS, spread on glass slides, air dried and stored at 4°C until use. Acrosome staining was then performed: after rehydrating the samples in PBS for 5 min at room temperature, lectin-PNA-Alexa Fluor 488 conjugates (50 µg/mL in PBS, Molecular Probes, Invitrogen, Cergy Pontoise, France) were added for 30 min at 37°C, then slides were washed for 5 min in PBS and mounted with coverslips using Vectashield mounting medium with DAPI (Vector, AbCys, Paris, France) to stain the sperm nuclei. Lectin-PNA binds to sugar moieties of the outer acrosomal membrane, thus only staining the sperm cells that did not undergo the acrosome reaction. Only cells showing a full acrosome were considered as positive, and the % of acrosome-reacted gametes was evaluated on at least 200 cells per slide and 3 different individuals were used for each condition.

### Sperm morphology

After determination of the acrosomal status, slides were examined using a phase contrast microscope and sperm cells were classified into four categories: normal sperm, sperm with a hairpin-shaped flagellum, sperm with an abnormally-angulated flagellum, and broken sperm cells. A minimum of 200 cells was counted per slide and 3 different individuals were used in each condition.

### Mayer's haematoxylin staining for testicular histology and elongated nuclei counts

Paraffin sections (5 µm) of testis from *wt* and *lxrα;β−/−* 4-month-old mice, fed with control or HCD, were stained by immersion for 30 sec in Mayer's haematoxylin. Slides were rinsed for 5 min in running water and then mounted using cytoseal 60 as a mounting medium (Electron Microscopy Sciences, Hatfield, USA). Elongated nuclei corresponding to spermatids or spermatozoa were then counted in a minimum of ten tubules per individual, with 3 different individuals in each condition.

### Oil red O staining

Cryosections (7 µm) were stained with Oil Red O (Sigma) as previously described in [12], a method commonly used to stain neutral lipids (triglycerides and cholesteryl esters).

### Intra-testicular testosterone level measurements

Testosterone levels were measured with the direct Elisa Kit “The EiAsy™ Way Testosterone” (Diagnostics Biochem Canada Inc.). Briefly, ¼ of testis were crushed in 400 µl PBS-BSA1%. Extracts were diluted to 1/60 and 1/100 before testosterone was determined using the kit. Protein quantities were determined on the same sample using BioRad protein Assay so as to express results in ng of testosterone per mg of protein. Results are expressed as the mean ± SEM of duplicate points from 3 different individuals in each condition.

### Immunohistochemistry

Caput epididymides were fixed in 4% paraformaldehyde (Sigma) in PBS and imbedded in paraffin. Seven-micrometer-thick paraffin sections were mounted on Superfrost® glass slides and then deparaffinized with Histoclear for 40 min (National Diagnostic, Merck Eurolab, Fontenay-sous-Bois, France), rehydrated through a graded series of ethanol solutions, and finally rinsed in distilled water. Endogenous peroxidases were inhibited (30 min in 0.3% H_2_O_2_ – Sigma – in water) and sections blocked in PBS-bovine serum albumin (BSA) 1% (w/v, Euromedex, Mundolsheim, France) for 30 min. Rabbit-polyclonal anti-cav-1 (1/5000, Sigma), anti F4/80 (1/100, macrophage marker, Novus Biologicals, Interchim, Montluçon, France), anti-matrix-metalloprotease-9 (1/100, Novus Biologicals), anti-smooth muscle α-Actin (1/5000, Epitomics, Euromedex, Mundolsheim, France), or anti-CD68 (1/50, Novus Biologicals) diluted in PBS-BSA 0.1% w/v were incubated overnight at 4°C. Sections were washed 5 min in PBS and incubated 1 hr with biotin-SP conjugated AffiniPure goat anti-rabbit IgG (H+L) antibodies (1/500 in PBS-BSA 0.1% w/v, Jackson Immuno- research, Immunotech, Marseille, France). After a wash in PBS, sections were incubated 30 min with peroxidase-conjugated streptavidin (1/500 in PBS, Jackson Immunoresearch). Color was developed with the Vector NovaRED substrate kit for peroxidase (Vector). Unless indicated, slides were counterstained with Haematoxylin QS (Vector), dehydrated and then mounted with Cytoseal 60 mounting medium (Electron Microscopy Sciences, Hatfield, USA) before observation.

### Western Blots

Proteins were extracted from liquid nitrogen frozen epididymal tissues stored at −80°C until use. Briefly, tissues were homogenized in HEPES 20 mM, NaCl 0.42 M, MgCl_2_ 1.5 mM, EDTA 0.2 mM, NP40 1%, phenylmethylsulfonyl fluoride 1 mM, Na_3_VO_4_ 0.1 mM, NaF 0.1 mM and complete 1× (Roche Diagnostics, Meylan, France). Lysates were centrifuged at 4°C for 15 min at 15000 g. Forty µg of total proteins were subjected to denaturing SDS-polyacrylamide gel electrophoresis and transferred on nitrocellulose membrane (Hybond ECL, Amersham Biosciences, Villejuif, France). Blots were blocked with Tris Buffered Saline (TBS) (Tris 50 mM, NaCl 150 mM, pH 7.6) containing 10% w/v low fat dried milk and 0.1% v/v Tween 20, and probed overnight at 4°C with anti-β actin (1/5000, Sigma, used as a loading control), anti-cav-1 (1/5000, used as a marker of smooth muscle cell function), anti-smooth muscle α-Actin (1/10000, used as a marker of smooth muscle cell structure) or anti-CD68 (1/500, used as a foam cell marker) diluted in 10% w/v low fat dried milk/0.1% v/v Tween-20/TBS, or anti-Mmp9 (1/1000, Abcam, Cambridge, UK, a metalloprotease produced by migrating smooth muscle cells in pathological situations such as atherosclerosis) diluted in 3% w/v low fat dried milk/0.1% v/v Tween-20/TBS. Secondary antibody was a goat anti-rabbit horseradish peroxidase-conjugated (1/5000, Amersham Biosciences), detected by the “ECL Western Blotting Detection” kit (Amersham Biosciences) on hyperfilms (Amersham Biosciences). Densitometric analyses were made with “Quantity one” software (Biorad).

### Statistical analysis

To determine whether differences were statistically significant, Student's t test was performed, using a two-tailed distribution. A *p*-value of 0.05 or less was considered to be statistically significant.

## Results

### Post-testicular infertility induced in high-cholesterol fed young *lxrα;β−/−* animals

**Figure 1:**
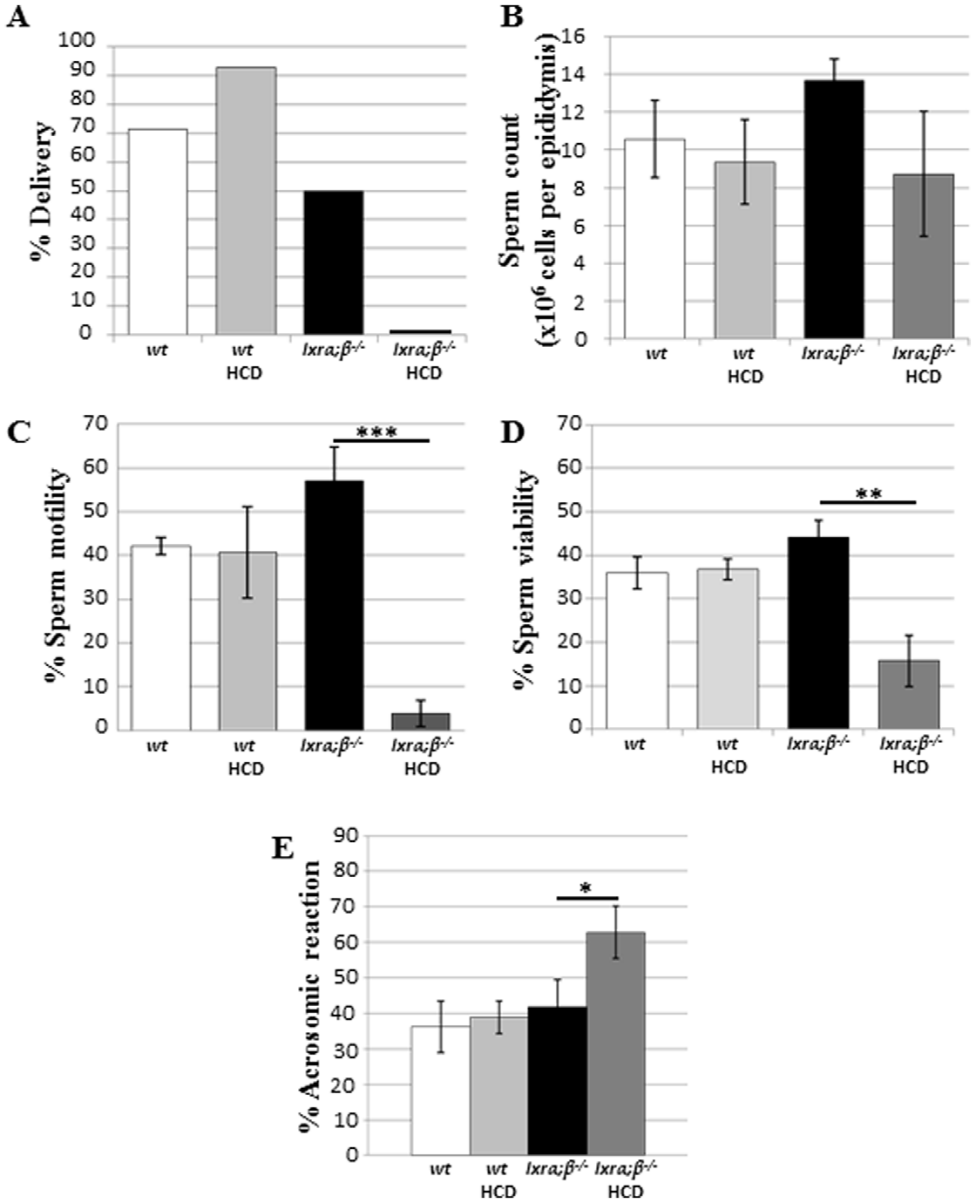
Post-testicular infertility induced in high-cholesterol diet-fed young *xrα;β−/−l* animals. (**A**) Delivery percentage obtained for 4-month-old *wt* or *lxrα;β−/−* male mice (n = 6 for each group), each mated with 2 SWISS *wt* females. Animals were fed for 4 weeks either a control or high-cholesterol diet (HCD) containing 1.25% cholesterol. (**B**) Number of cauda epididymidis retrieved spermatozoa per epididymis. Bar graphs display means ± SEM, n = 6. (**C**) Total motility percentage (Computer Assisted Sperm Analysis) of cauda epididymidis-retrieved spermatozoa. Bar graphs display means ± SEM, n = 3. (**D**) Viability percentage of cauda epididymidis-retrieved spermatozoa. Bar graphs display means ± SEM, n = 6. (**E**) Percentage of spontaneously acrosome-reacted sperm cells determined on cauda epididymidis-retrieved spermatozoa. Bar graphs display means ± SEM, n = 6, *p<0.05, **p<0.01, ***p<0.001.

Fertility and sperm parameters of 4-month-old *wt* or *lxrα;β−/−* male mice fed a control or lipid-enriched diet (thereafter referred to as High-Cholesterol Diet (HCD)) for 4 weeks are presented in Fig. 1. The delivery rate (percentage of mated females giving birth to live offspring) indicated that HCD-fed *lxrα;β−/−* male mice were totally infertile, as no gestating females and, consequently, deliveries were observed (Fig. 1A). The presence of copulatory plugs was similar between different groups (not shown), indicating that infertility was not due to an impaired mounting behavior. Sperm counts, following sperm retrieval from the cauda epididymides, did not differ between *wt* and *lxrα;β−/−* mice fed the control diet or HCD (Fig. 1B), suggesting that infertility was not related to testicular spermatogenic efficiency. Sperm parameter analyses revealed that HCD provoked dramatic decreases in sperm motility (Fig. 1C, 93% decrease; p<0.001) and viability (Fig. 1D, 64% decrease; p<0.01) in the *lxrα;β−/−* mice compared with controls. Measurements of the acrosomal status revealed that HCD also provoked a premature loss of acrosome in *lxrα;β−/−* males (Fig. 1E). Furthermore, the analysis of sperm morphology showed that HCD caused a significant increase in the percentage of broken cells only in *lxrα;β−/−* mice (Table 1).

**Table 1:** Percentages of morphologically normal and abnormal sperm cells.

Group	Normal	Hairpin	Angulated	Broken
*wt*	25.3±3.6	63.1±2.5	6.3±1.2	5.3±2.4
*wt* HCD	25.8±4.0	56.4±6.4	6.9±1.2	11±1.5
*lxrα;β−/−*	37.5±5.6	51.7±6.6	7.6±0.1	3.2±1.1
*lxrα;β−/−* HCD	19.2±8.5	49.6±6.7	6.8±1.2	24.4±1.5^a^^,^ b

Results are presented as means ± SEM.

a = p≤0.05 compared to *lxrα;β−/−*;

b = p≤0.05 compared to *wt* on a high-cholesterol diet.

### Testicular sperm production was not affected by the high-cholesterol diet

**Figure 2:**
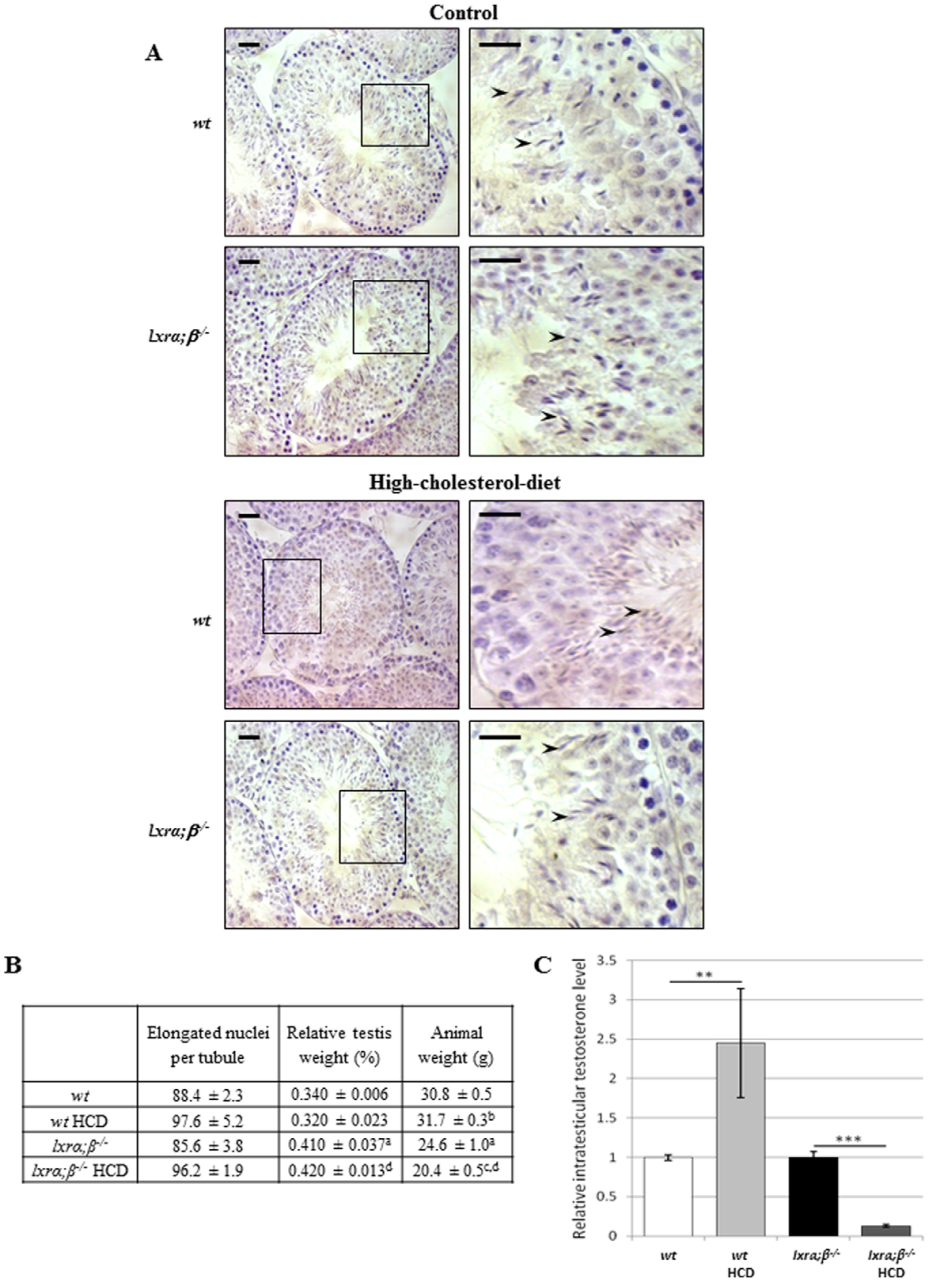
Testicular function in high-cholesterol diet fed young mice. **A.** Paraffin sections of testis from *wt* (upper panel) and *lxrα;β−/−* (lower panel) 4-month-old mice fed a control and high-cholesterol diet, respectively, were stained by Mayer's haematoxylin. A higher magnification is presented for each condition. Scale bars represent 20 µm. **B.** Elongated nuclei corresponding to spermatids or spermatozoa (arrowheads) were counted in a minimum of ten tubules per individual, with 3 different individuals in each condition. Results are presented in the table below, together with relative testis weight (% of the whole body) and animal weights (g), and are expressed as mean ± SEM. a = p<0.05 compared to *wt*; b = p<0.05 compared to *wt*; c = p<0.05 compared to *lxrα;β−/−*; d = p<0.05 compared to *wt* high-cholesterol diet. **C.** Intratesticular testosterone quantification of control and high-cholesterol diet-fed 4-month-old wild type and *lxrα;β−/−* animals. Bar graphs are expressed as a normalized value of the expression level in high-cholesterol diet-fed animals versus an arbitrary value of 1 in the control-fed animals. Each value is the mean ± SEM using protein level as internal standard. Bar graphs display means ± SEM, n = 5. **p<0.01; ***p<0.001.

The above-presented results on sperm properties strongly suggested epididymal defects. Thus, testicular sperm production was evaluated to test this hypothesis. Haematoxylin staining did not reveal any dramatic HCD-induced histological perturbation of the testis as shown in Fig. 2A. The sperm production efficiency was not affected as evidenced by counts of cauda epididymidis-retrieved gametes (see above and Fig. 1B). This data was corroborated by counts of elongated nuclei in the seminiferous tubules (corresponding to elongated spermatids or spermatozoa, see arrowheads in the higher magnification photomicrograph of Fig. 2A) that were similar under all conditions (Fig. 2B). Furthermore, HCD did not affect relative testicular weights; they even increased for the LXR-deficient males when expressed as a percentage of the body weight (however, this increase was related to a decrease in animal weight associated with the metabolic effects of the feeding regimen, see Fig. 2B). In summary, these data confirmed that the efficiency of testicular sperm production was not affected by diet. To evaluate whether the endocrine function of the testis was altered by diet, we measured testicular testosterone levels and found them significantly reduced in *lxrα;β−/−* animals fed HCD compared to *lxrα;β−/−* animals fed the control diet (Fig. 2C). In *wt* animals, the diet induced a significant increase in intra-testicular testosterone level (Fig. 2C).

### High-cholesterol diet altered the caput epididymal epithelium in a segment and cell-specific manner in young *lxrα;β−/−* animals

**Figure 3:**
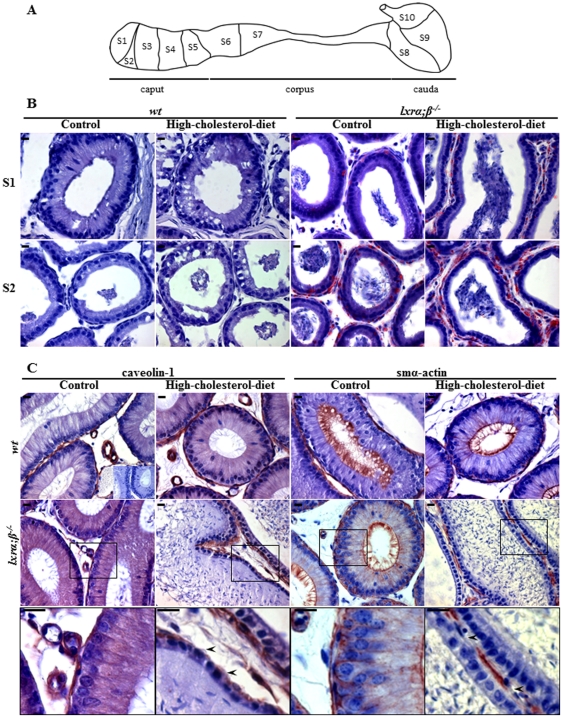
Effect of high-cholesterol diet on caput epididymidis tissue in a segment and cell-specific manner in young *lxrα;β−/−* animals. (**A**) Schematic representation of the adult mouse epididymis. Caput, corpus and cauda designate the 3 main regions of the mammalian epididymis from its proximal extremity connected to the efferent duct to its distal extremity connected to the vas deferens (not illustrated). Sub-regions 1 to 10 identify the various epididymis segments that are anatomically separated by connective tissue septa [33]. (**B**) Oil Red O staining of segments 1 (S1) and 2 (S2) caput epididymidis of 4-month-old *wt* or *lxrα;β−/−* fed for 4 weeks a control diet or high-cholesterol diet (scale bars represent 10 µm, n = 3). (**C**) Caveolin-1 and smα-actin immunoperoxidase stainings in segment 1 caput epididymidis from *wt* and *lxrα;β−/−* mice at 4 months of age fed for 4 weeks a control diet or high-cholesterol diet. The lower panel displays higher magnifications of the region included in the box of the second panel (*lxrα;β−/−* animals). Inset represents negative control. Scale bars represent 10 µm, n = 3.

To test the hypothesis that sperm defects recorded in LXR-deficient males on HCD were mainly caused during post-testicular maturation, histological and immunohistological aspects of the epididymides of these animals were compared with those of LXR-deficient males fed a control diet and wild type males fed either a control or high-cholesterol diet (Fig. 3). Caput epididymidal cryosections were stained with Oil Red O to evaluate neutral lipid accumulations. Lipid droplets were clearly visible in segments 1 and 2 of *lxrα;β−/−* mice (see Fig. 3A for classical mouse epididymal segmentation), and they were significantly increased in HCD-fed animals (Fig. 3B). Only segments 1 and 2 are illustrated, because there was no epididymal macroscopic change distal to segment 2. These accumulations were not observed in *wt* animals and were essentially peritubular involving smooth muscle cells (SMC) lining the epididymal tubules (see Fig. S1 for higher magnifications). In accordance with increased tissue lipid deposition HCD provoked a large elevation of plasma LDL in both *wt* and *lxrα;β−/−* mice (Fig. S2A). In agreement with a SMC localization of lipid droplets, immunodetection with functional and structural SMC markers (respectively, cav-1 for caveolin-1 and smα-actin for smooth muscle alpha-actin) revealed a decrease of both markers in peritubular SMC of the caput segment 1 of HCD-fed *lxrα;β−/−* mice only (Fig. 3C, arrowheads in higher magnification microphotographs of the lower panel). This phenotype was also observed in segment 2 (Fig. S3) but not in the more distal segments of the epididymis (not shown). The defects recorded in caput segments 1 and 2 in HCD-fed young LXR-deficient males were also associated with a reduction in epithelial height (6.6±0.7 µm vs. 20.9±1.9 µm, p<0.05) and a loss of luminal cilia, normally visible as a non-specific signal with the anti-smα-actin immunostaining (see Fig. 3C), two known characteristics of the epididymal phenotype observed in old *lxrα;β−/−* animals [10], [11].

### High-cholesterol diet and ageing alter proximal caput epididymis SMC markers in LXR-deficient male mice

**Figure 4:**
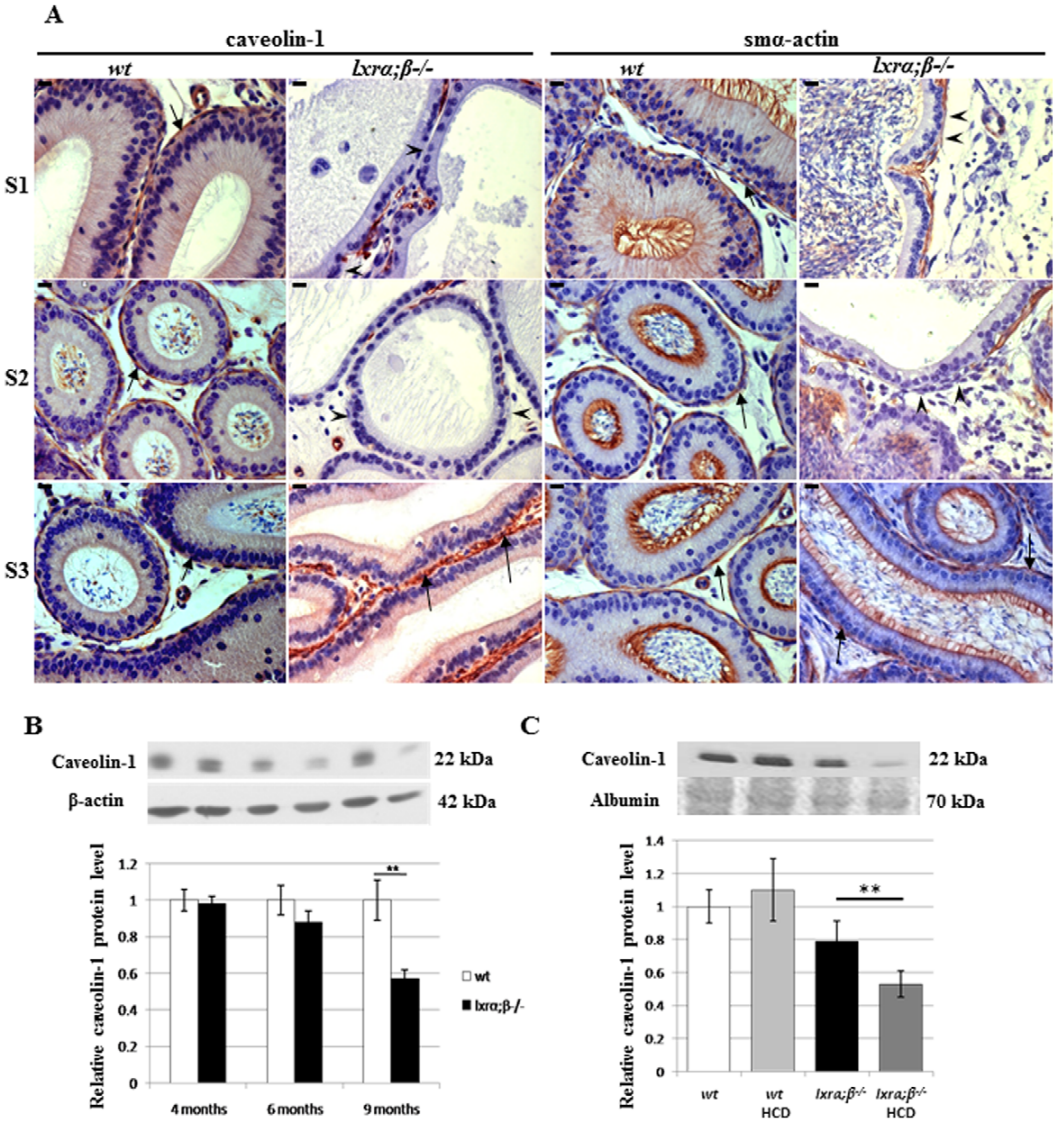
Modification of proximal caput epididymal SMC markers in relation to age and diet. (**A**) Caveolin-1 and smα-actin immunoperoxidase staining in segment 1 (S1), segment 2 (S2) and segment 3 (S3) of the caput epididymidis from *wt* and *lxrα;β−/−* mice at 9 months of age. Arrows indicate the positive staining and arrowheads indicate places where the staining is not present in S1 and S2 of the *lxrα;β−/−* mice. Scale bars represent 10 µm, n = 3. (**B**) Relative levels of caveolin-1 protein in the caput epididymidis from *wt* and *lxrα;β−/−* mice at 4, 6 and 9 months of age. Bar graphs display means ± SEM using β-actin as an internal standard for quantification, n = 3, **p<0.01. (**C**) Relative protein levels of caveolin-1 in the caput epididymidis of 4-month-old *wt* or *lxrα;β−/−* animals fed a control or high-cholesterol diet for 4 weeks. Bar graphs display means ± SEM using albumin (Ponceau red) as an internal standard for quantification, n = 3, **p<0.01.

SMC markers were investigated in infertile 9-month-old *lxrα;β−/−* animals fed a standard diet. Similarly to what was observed in younger animals fed a HCD, it appeared that the characteristic peritubular staining of cav-1 and smα-actin (Fig. 4A, arrows) also decreased in caput epididymidis segments 1 and 2 (Fig. 4A, arrowheads), whereas no abnormality was seen in posterior segments illustrated here only for segment 3 (Fig. 4A, arrows). The loss of luminal cilia was also visible in these tissues. The quantitative decrease of caveolin-1 started as early as 6 months of age, although not in a significant manner, and became significant in the caput epididymidis of 9-month-old animals as revealed by immunoblotting (Fig. 4B). This decrease, occurring in older LXR-deficient animals on a standard diet, is triggered by HCD already in 4-month-old *lxrα;β−/−* animals (Fig. 4C). Overall, these results show that 4-month-old *lxrα;β−/−* animals after 4 weeks on HCD display a phenotype normally occurring in ageing males. A decreased accumulation of cav-1 level in the caput epididymidis can thus be considered as a molecular marker of the SMC degenerative phenotype.

### Peritubular SMC of the epididymis transdifferentiate into foam cells

**Figure 5:**
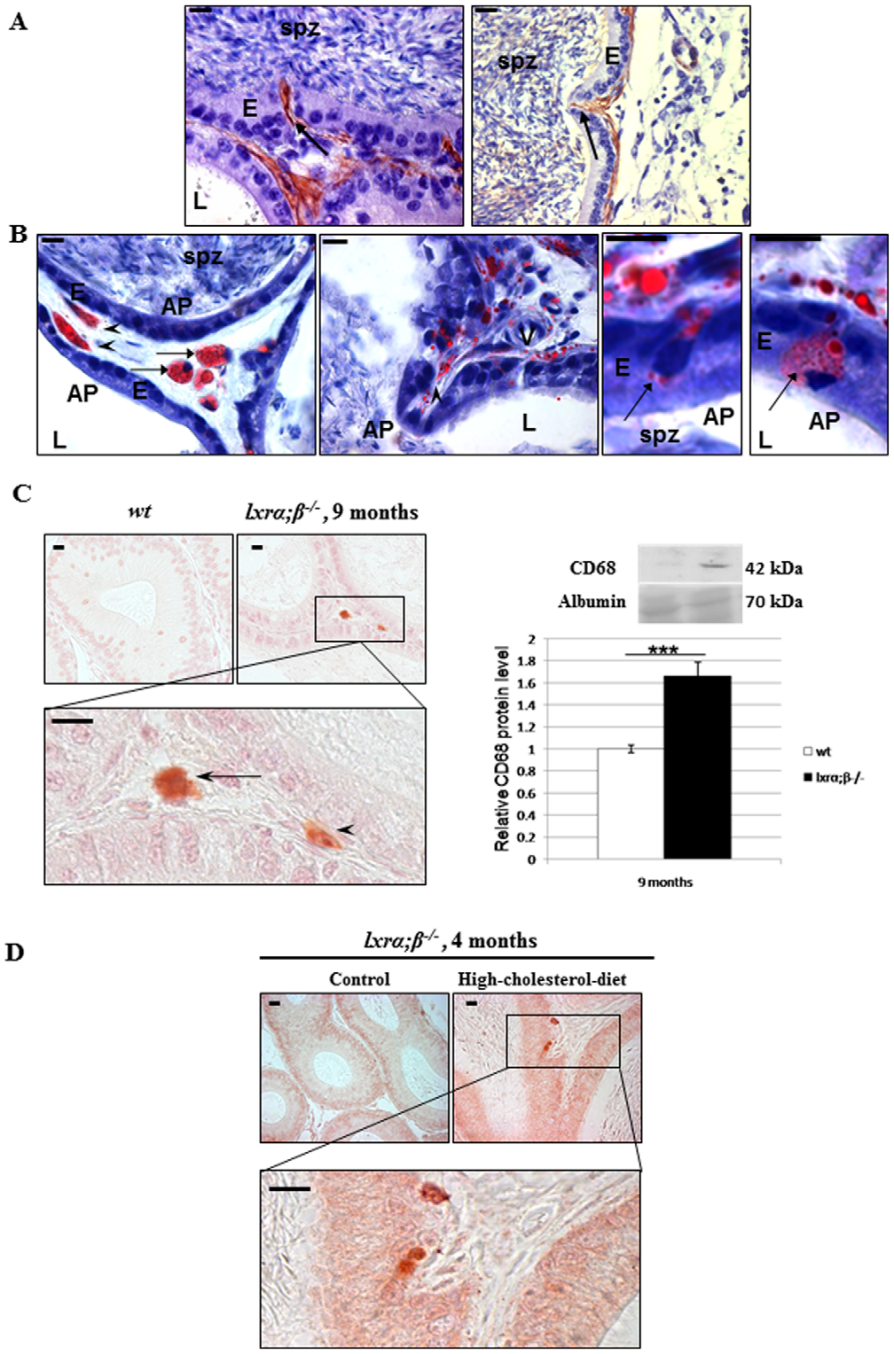
Transdifferentiation of epididymal peritubular SMC into foam cells in 9-month-old *lxrα;β−/−* animals as well as in high-cholesterol diet-fed 4-month-old *lxrα;β−/−* animals. (**A**) Caveolin-1 (left panel) and smα-actin (right panel) immunoperoxidase staining in segment 1 of the caput epididymidis from 9-month-old *lxrα;β−/−* mice showing smooth muscle invaginations (arrows) through the epididymal epithelium. Scale bars represent 10 µm. (**B**) Oil Red O stains of segment 1 of the caput epididymidis of 9-month-old *lxrα;β−/−* mice show neutral lipid accumulation in smooth muscle cells (arrowheads), foam cells and protruding cells (arrows). Scale bars represent 10 µm, n = 3. In A and B, abbreviations are: AP = apical pole, E = epithelium, L = lumen, spz = spermatozoa, V = blood vessels. (**C**) Immunoperoxidase staining (left panel) of CD68 in 9-month-old *wt* (upper left picture) and *lxrα;β−/−* mice, not counterstained. Scale bars represent 10 µm, the arrow indicates a foam cell and arrowhead indicates a transdifferentiating SMC. Relative protein levels (right panel) of CD68 in 9-month-old *wt* and *lxrα;β−/−* mice, n = 3, ***p<0.001. (**D**) Immunoperoxidase staining of CD68 in 4-month-old *lxrα;β−/−* mice fed a control (upper left picture) or high-cholesterol diet, not counterstained. Scale bars represent 10 µm, n = 3.

Looking closer at the caput epididymidis phenotype, we observed in several locations peritubular cells involved in an infiltration-like process through the epididymal epithelium. These cells were SMC as they were positively stained with anti-cav-1 and anti-smα-actin (Fig. 5A, arrows). Concomitantly, oil-red-O staining of cryosections from the same samples revealed that the peritubular accumulations of lipid droplets concerned SMC fusiform cells that were also present in the infiltrations (Fig. 5B, arrowheads). Since these features resembled alterations seen in atherosclerotic vascular cell walls, we tested for the expression of CD68 and Mmp-9, respectively, which are markers of foam cells [15] and extracellular matrix degradation [16], in proximal caput epididymidis of both 9-month-old LXR-deficient animals on a control diet and 4-month-old *lxrα;β−/−* males on HCD for 4 weeks. We observed that an anti-CD68 antibody clearly identified peritubular foam cells in 9-month-old *lxrα;β−/−* proximal epididymis (Fig. 5C, left). A quantitative western blot evaluation of CD68 showed its accumulation in caput epididymidis protein extracts of 9-month-old *lxrα;β −/−* animals (Fig. 5C, right). CD-68 positive cells were also present in the peritubular compartment of 4-month-old *lxrα;β−/−* males fed the HCD for one month (Fig. 5D).

**Figure 6:**
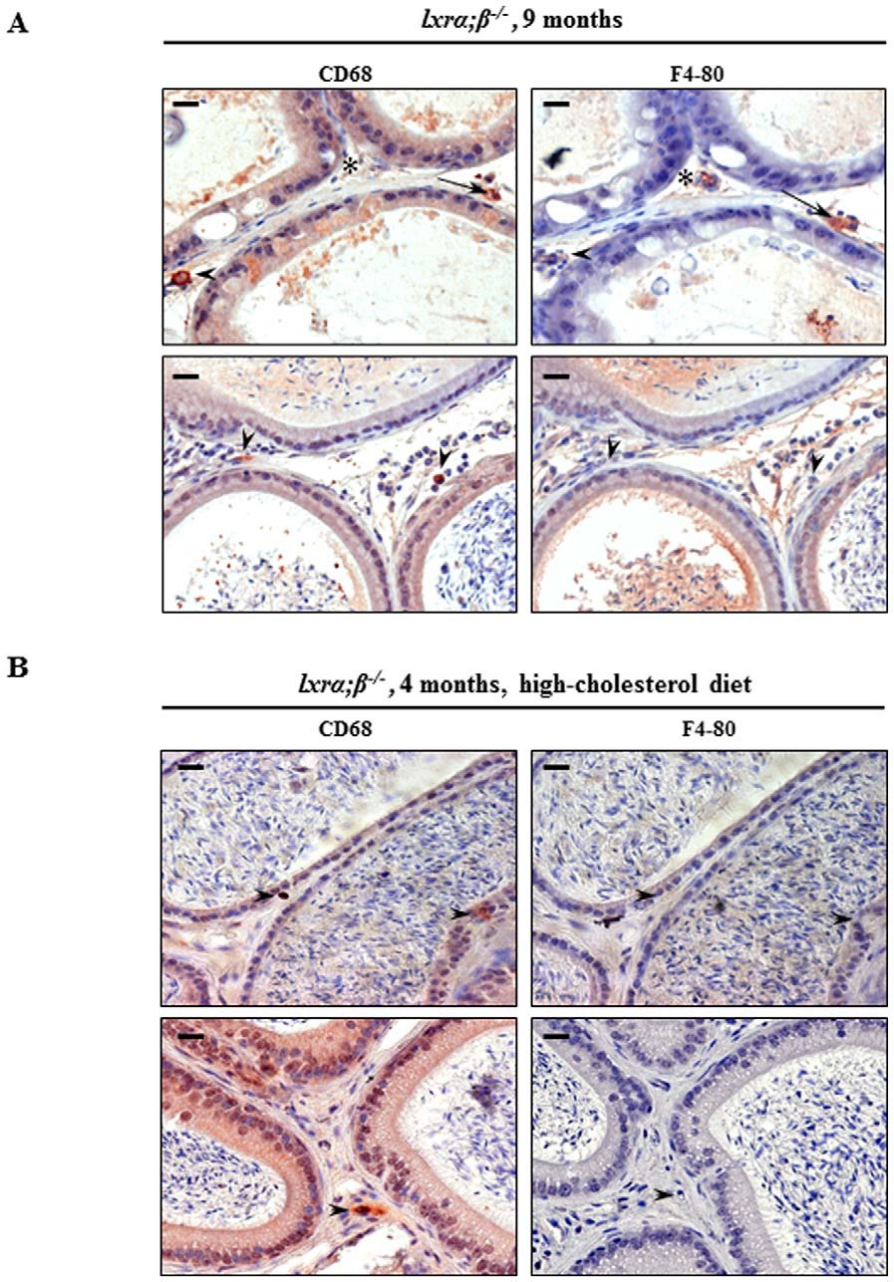
Foam cells originate either from the transdifferentiation of epididymal peritubular SMC or from macrophages. Immunoperoxidase staining of CD68 and F4/80 in adjacent paraffin sections of (**A**) 9-month-old *lxrα;β−/−* mice and (**B**) 4-month-old *lxrα;β−/−* mice fed 1 month with high-cholesterol diet. Positive cells for CD68 only are indicated by an arrowhead, positive cells for F4/80 only are indicated by a star (*) and positive cells for both markers are indicated by an arrow, scale bars represent 10 µm, n = 3.

To define whether the CD68 positive cells could arise from circulating macrophages, we performed immunoperoxidase staining for a specific macrophage marker, F4/80, and for CD68 on adjacent sections of caput epididymidis from 9-month-old *lxrα;β−/−* animals (Fig. 6A). Three types of cells were represented, i.e. either cells exclusively positive for CD68 (arrowheads) or F4/80 (stars), respectively, or cells positive for both markers (arrows). The foam cells in caput epididymidis from 9-month-old *lxrα;β −/−* males thus originated from both SMC transdifferentiation and macrophage infiltration. The same experiments were performed in young *lxrα;β −/−* males on HCD (Fig. 6B); no double-stained cells were observed, indicating that the foam cells had originated from transdifferentiating SMC only.

**Figure 7:**
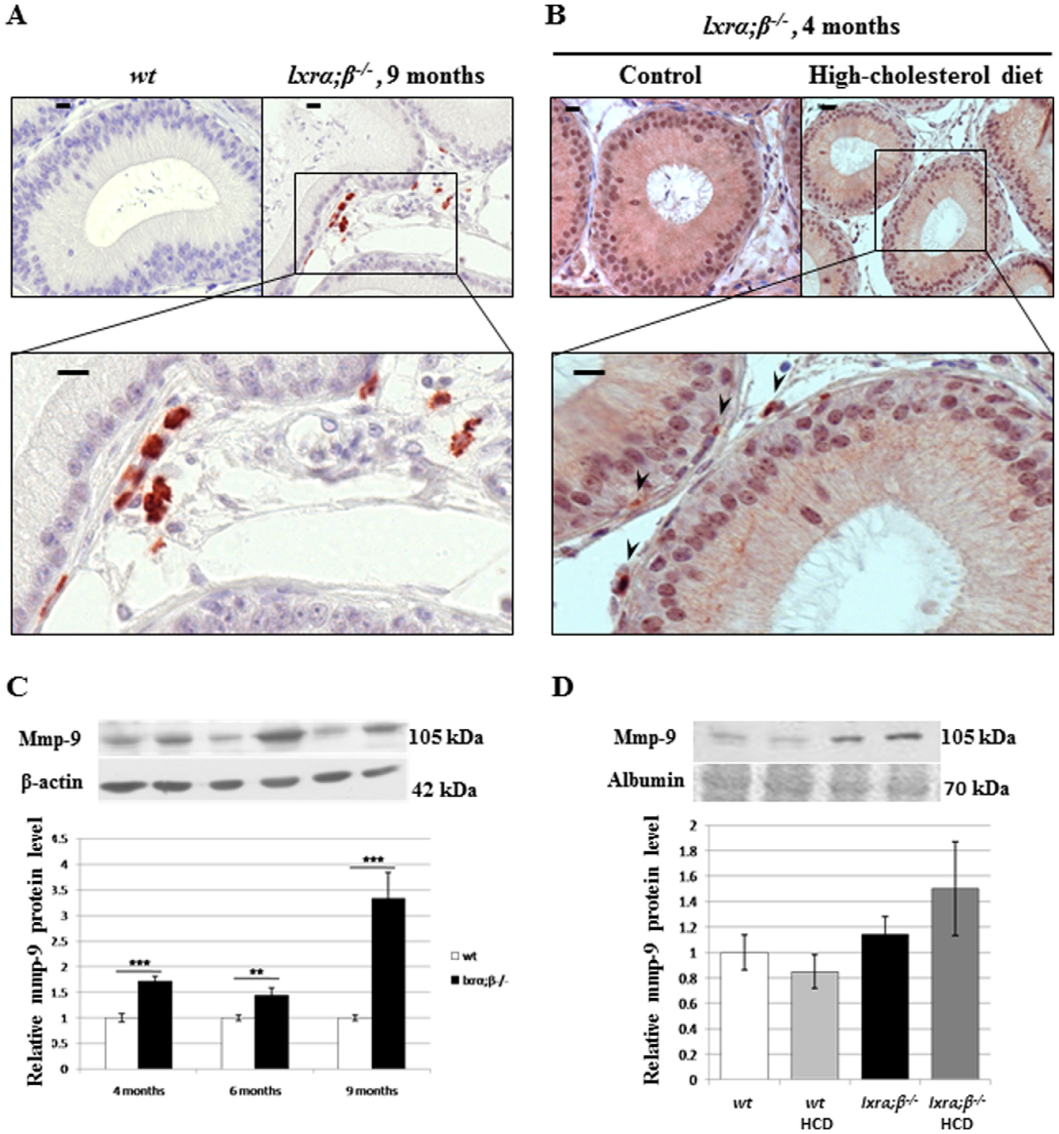
Transdifferentiation of SMC is associated with matrix-metalloprotease-9 (Mmp-9) expression. Immunoperoxidase staining of Mmp-9 in caput epididymidis segment 1 of (**A**) 9-month-old *wt* (upper left picture) or *lxrα;β−/−* mice and (**B**) 4-month-old *lxrα;β−/−* mice fed 1 month with the control diet (upper left panel) or the high-cholesterol diet. Arrowheads indicate Mmp-9 positive cells in 4-month-old *lxrα;β−/−* mice. Scale bars represent 10 µm, n = 3. (**C**) Relative Mmp-9 protein levels in the caput epididymidis from *wt* and *lxrα;β−/−* mice at 4, 6 and 9 months of age. Bar graphs display means ± SEM using β-actin as an internal standard for quantification, n = 3, **p<0.01; ***p<0.001. (**D**) Relative protein levels of Mmp-9 in the caput epididymidis of 4-month-old *wt* or *lxrα;β−/−* animals fed a control or High-cholesterol diet for 4 weeks. Bar graphs display means ± SEM using albumin (Ponceau red) as an internal standard for quantification, n = 3.

Matrix degradation processes accompanied the presence of foam cells as we could observe considerable accumulation of Mmp-9 in cells surrounding the tubules in the caput epididymidis from 9-month-old LXR-deficient animals (Fig. 7A). The expression levels of Mmp-9 in caput epididymidis protein extracts of 9-month-old LXR-deficient animals was highly increased compared to 4-and 6-month-old animals (Fig. 7C), in accordance with the loss of SMC seen by the drop in cav-1 levels at the same age. Mmp-9 showed a tendency to increase in caput epididymidal protein extracts of young *lxrα;β−/−* males on HCD (Fig. 7D), as confirmed by immunoperoxidase staining (Fig. 7B).

## Discussion

This work indicates that Liver-X-Receptors are key actors in post-testicular sperm maturation processes in the proximal epididymis. These receptors are involved in the maintenance of (i) epididymal cholesterol balance and (ii) epididymal structure, especially in animals fed a lipid-enriched diet. In addition to the ABCA1-mediated accumulation of cholesteryl esters in apical cells of the *lxrα;β−/−* proximal epididymal epithelium reported previously [11], we showed here that smooth muscle cells surrounding the epididymal tubules also had impaired function in LXR-deficient animals. *Lxrα;β−/−* animals progressively lose SMC function in the proximal caput epididymidis segments 1 and 2 as illustrated by the decrease of caveolin-1, a marker of SMC contractility [17], suggesting that peristaltic contractions and, consequently, sperm progression in the lumen of the tubule may be impaired, perturbing the finny orchestrated process of spermatozoa epididymal maturation. This is in agreement with our earlier observation that the tubules of the ageing *lxrα;β−/−* animals are filled with amorphous material [10]. The participation of cav-1 in intracellular cholesterol movements and efflux to extracellular acceptors [18], [19] could explain CE accumulations as cav-1 decreases in epididymal SMC. The description that a dominant negative mutation of cav-1 leads to neutral lipid storage in lipid droplets supports this hypothesis [20]. SMC within the proximal caput epididymidis of *lxrα;β−/−* animals were also found to transdifferentiate into foam cells that migrated across the epididymal epithelium towards the lumen. These cells have acquired known characteristics of foam cells, i.e. cytoplasm filled with lipid droplets and CD68 overexpression. This is quite similar to the situation encountered in mouse aortic SMC, in which a decrease in smα-actin, lipid droplet accumulation and an increase in CD68 are markers of their transdifferentiation into a macrophage-like state, a critical step in the atherosclerotic process [15]. SMC have also been described as a source of foam cells in human coronary atherosclerosis [21] and in atheromatous lesions of human aortas [22]. In our epididymal context, the CD68-positive cells were shown to have two potential origins in 9-month-old males, as some were also F4/80 positive, a macrophage specific marker (Fig. 5), suggesting macrophage infiltration from blood vessels. In the 4-month-old *lxrα;β−/−* males fed the HCD, only CD68-positive cells were observed (Fig. 6B), perhaps indicating two different kinetics of the sclerotic development. This was associated with a significant increase in Mmp-9 levels that was more obvious in the 9 month old *lxrα;β−/−* males than in younger animals on HCD. The inflammation process may be more involved in the development of the pathology in older males under control diet, whereas SMC transdifferentiation may be more relevant in younger HCD-fed animals, a hypothesis that will need further testing. In human and mouse atherosclerotic lesions, Mmp-9 has been shown to participate in SMC migration [23]. The finding of Mmp-9 overexpression in our study agreed with the observation, that LXR down-regulated Mmp-9 expression in mouse peritoneal and bone-marrow-derived macrophages [16]. Our data regarding the LXR-mediated effect on SMC also agrees with reports showing that a lack of LXR impairs mouse uterine contractility [24]. Therefore, SMC of the proximal caput epididymidis appear to be another target of LXR action and loss of peritubular SMC in *lxrα;β−/−* animals has been associated with reduction in epithelium height, particularly visible in the two first segments of 9-month-old *lxrα;β−/−* mice or younger *lxrα;β−/−* animals fed a lipid-enriched diet. SMC do not appear to undergo apoptosis, as they were TUNEL negative [11]. It is thus likely, that their migration towards the epididymal lumen caused the structural changes in the epithelium. As found in aortic SMC transdifferentiation after cholesterol loading [15], we showed here that the ageing *lxrα;β−/−* epididymal phenotype could be triggered in younger animals by feeding a cholesterol-enriched diet. Most importantly, the appearance of the phenotype was associated with complete male infertility, which was unlikely caused by direct effects of the diet on testicular functions. Indeed, normal testicular activity was supported by several observations: epididymal sperm counts were not different between *wt* and *lxrα;β−/−* animals independent of diet (Fig. 1B), testicular sperm production was similar in the four groups, and no decrease in testicular weight was noticed (Fig. 2B). The slight increase in relative testicular weight observed in *lxrα;β−/−* males is related to the global weight loss of the animals also reported in Fig. 2B. Testicular histology did not reveal any visible alterations, the opposite of what was seen in caput epididymidis, reinforcing the idea that the epididymis is affected early in a situation of cholesterol overload. This is supported by the results obtained for sperm morphology, sperm motility, sperm viability and acrosome integrity, that were severely impaired in high-cholesterol diet *lxrα;β−/−* animals. However, it is possible that at least a part of the impairment of sperm quality may be related to dysfunction in the last steps of spermatogenesis, the spermiogenesis process, a point that will need further investigation. Due to its similarities to atherosclerosis, we propose the observed phenotype to be referred to as “epididymosclerotic”.

Knowing that LXR deficiency somehow establishes a state of hypo-androgeny in aged animals [8], the observed decrease in intra-testicular testosterone levels in HCD-fed *lxrα;β−/−* animals came as no surprise. One might argue that this decrease was responsible for the epididymal phenotype triggered by HCD, because it is known that the epididymal physiology is largely independent of systemic androgens, i.e. androgens in the epididymis originate from the testis as a lumicrine factor. The generation and analysis of the PEARKO mouse model [25], in which androgen signaling had been altered in accessory sex organs including the prostate and the epididymis, showed that the animals presented with slight histological defects only in corpus and cauda epididymis, which was the opposite to our model (i.e., caput-restricted defects). More recently, two groups have reported new informative data concerning the roles of androgens in the proximal caput epididymidis via the generation of a selective knockout of the androgen receptor in the proximal caput epithelium [26], [27]. In both models, the knockout resulted in severe abnormalities of the initial caput epididymidis segment, leading to male infertility due to obstructive azoospermia, a situation that is again different to what we report here. The infertility in our *lxrα;β−/−* animals was mainly due to sperm impairment as attested by the high number of broken cells in the cauda epididymidis, even though the cauda sperm counts were similar, which also did not indicate an obstruction of the epididymal duct. The observed decrease in intratesticular testosterone rather resembles that of orchidectomy in the adult. In that case, it was shown that sperm cells lacked and that epididymal cells underwent apoptosis, something that was not observed in our animals (confirmed in [11]). Furthermore, orchidectomy-dependent epididymal alterations can be restored by testosterone replacement, except for the changes in the initial segment, and we previously demonstrated that testosterone replacement did not restore the epididymal phenotype of the *lxrα;β−/−* animals [10]. Data published by other groups also showed that a drop in intratesticular testosterone level as early as 2.5 months of age in *lxrα;β−/−* animals does not trigger any infertility at that age [8]. Moreover, it was also reported in 10-month-old wild type mice that testosterone levels similar to those recorded here had no noticeable effects on fertility [28]. Considering all these data, we cannot exclude a role of decreased testosterone levels in the epididymal phenotype. However, we show here the very peculiar sensitivity and the regionalized pro-inflammatory behavior of the proximal caput epithelium in response to a dietary cholesterol overload. The diet had the opposite effect in *wt* animals, i.e. it provoked a significant increase in intra-testicular testosterone levels (Fig. 2C). This could be explained by the ability of steroidogenic tissues to increase hormone production when the substrate, cholesterol from lipoproteins, is available in higher quantities, which is the case here (Fig. S2). It has previously been correlated with an increase in steroidogenic acute regulatory (StAR) mRNA and protein levels, the shuttle taking cholesterol in the mitochondrial matrix for steroid synthesis [29].

The infertility phenotype is associated to an impaired motility and the appearance of an increased number of broken sperm cells in *lxrα;β−/−* animals fed the HCD. It will thus be necessary to undergo *in-vitro* fertilization protocols in order to evaluate the real impact of these alterations on the fertilization capacities of the sperm cells. This will help determine whether the altered sperm functions rely on motility disorders, oocyte recognition and fertilization, or both. These aspects are currently under investigation.

This study highlights the sensitivity of the post-testicular compartment, i.e. the epididymis, with its maturing and stored pools of spermatozoa, to dietary cholesterol overload. It may help to understand human infertility in male dyslipidemic patients [3], [30], [31] and link paternal obesity to defective sperm parameters [4], [32]. This work clearly shows that the epididymis is an early target of lipid-induced infertility and that its function can be dramatically altered upon dietary cholesterol overload. Thus, the regulation of epididymal lipid and cholesterol homeostasis *via* LXR appears to be a critical factor in maintaining fertility in both ageing males and in younger males with lipid disorders.

## Supporting Information

Figure S1 **Neutral lipid accumulation in peritubular smooth muscle cells from LXR-deficient 4-month-old animals fed the control diet (upper panel) or with the high-cholesterol diet for 1 month (lower panel).** Oil red O staining of 7 µm cryosections revealing neutral lipids in red (triglycerides and cholesteryl esters). Highly lipid-loaded smooth muscle cells as well as protruding cells are visible in the lower panel. Scale bars represent 10 µm.
(DOC)

Figure S2 **Plasma LDL increases in high-cholesterol diet treated mice and in ageing** ***lxrα;β−/−*** **mice.** Plasma cholesterol, triglycerides, HDL and LDL concentrations were measured (as described in Text S1) in (**A**) *wt* and *lxrα;β−/−* 4-month old mice fed the control or the high-cholesterol diet and (**B**) *wt* and *lxrα;β−/−* ageing mice at 4, 6 and 8 months of age. Histograms are expressed as mean ± SEM, n = 3. _*_p<0.05; _**_p<0.01.
(DOC)

Figure S3 **Modification of caput epididymidal segment 2 SMC markers in high-cholesterol diet-fed 4-month-old lxrα;β−/− animals.** Caveolin-1 and smα-actin immunoperoxidase staining are decreased (arrowheads in the bottom panel microphotographs) in segment 2 (S2) of the caput epididymidis from *lxrα;β−/−* mice at 4 months of age after four weeks of high-cholesterol diet. Inset represents negative control, scale bars represent 10 µm, n = 3.
(DOC)

Text S1 (DOCX)
