# Supplementary figures and images for: Dietary Cholesterol-Induced Post-Testicular Infertility
Source: PLoS One. 2011 Nov 2;6(11):e26966. doi: 10.1371/journal.pone.0026966 (PMC3206870; doi:10.1371/journal.pone.0026966)

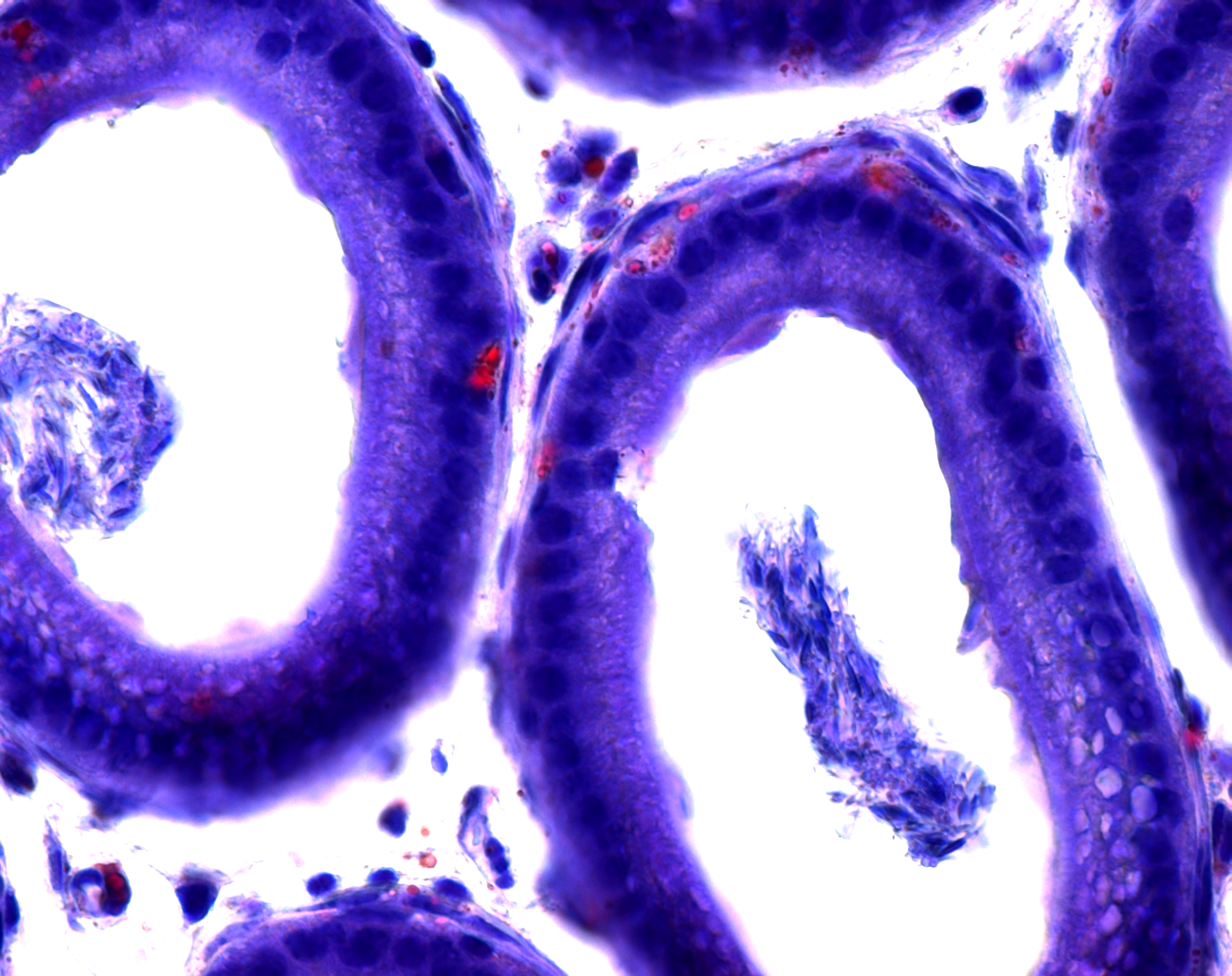

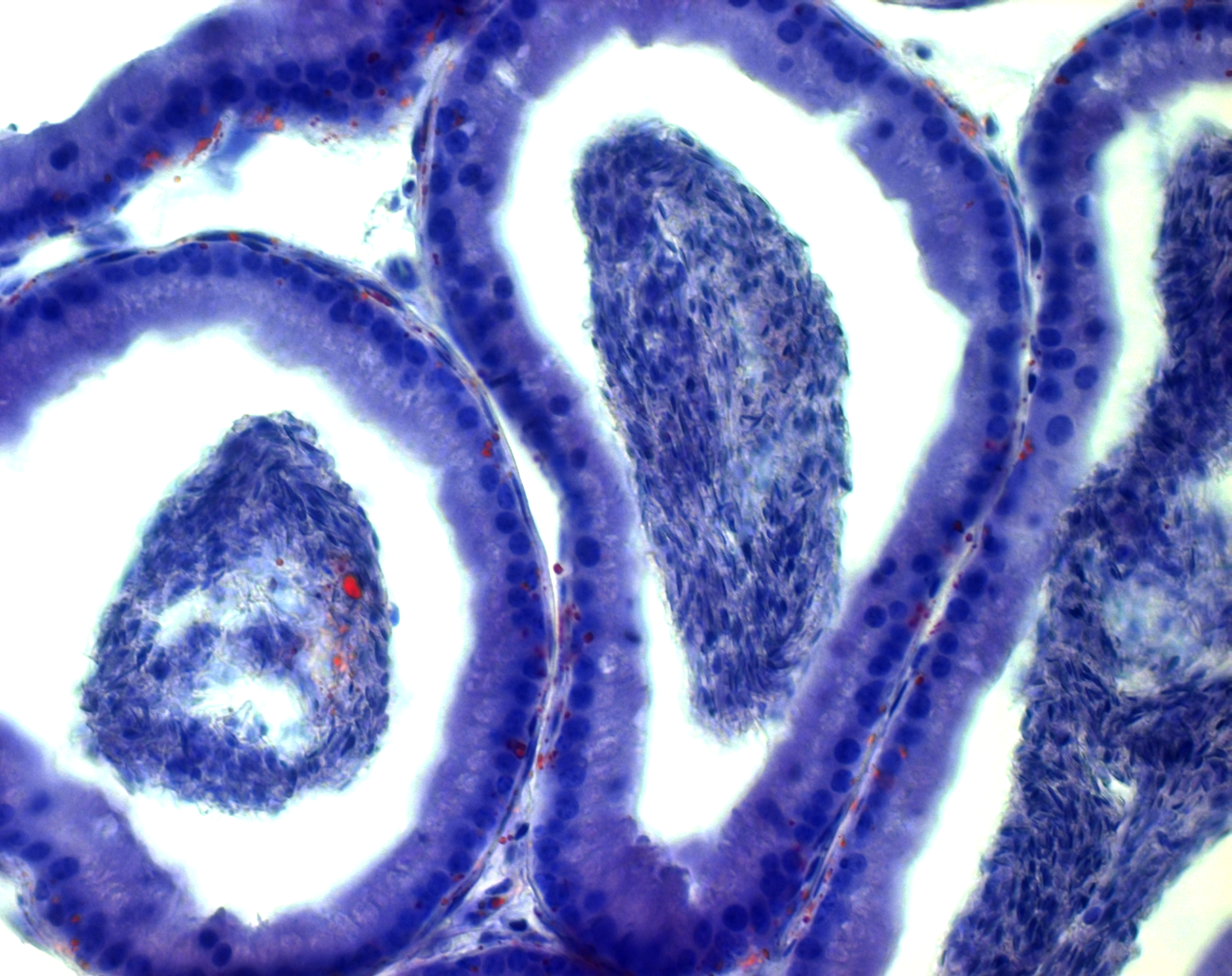
***lxrα;β-/-* 4 months of age, control diet.**


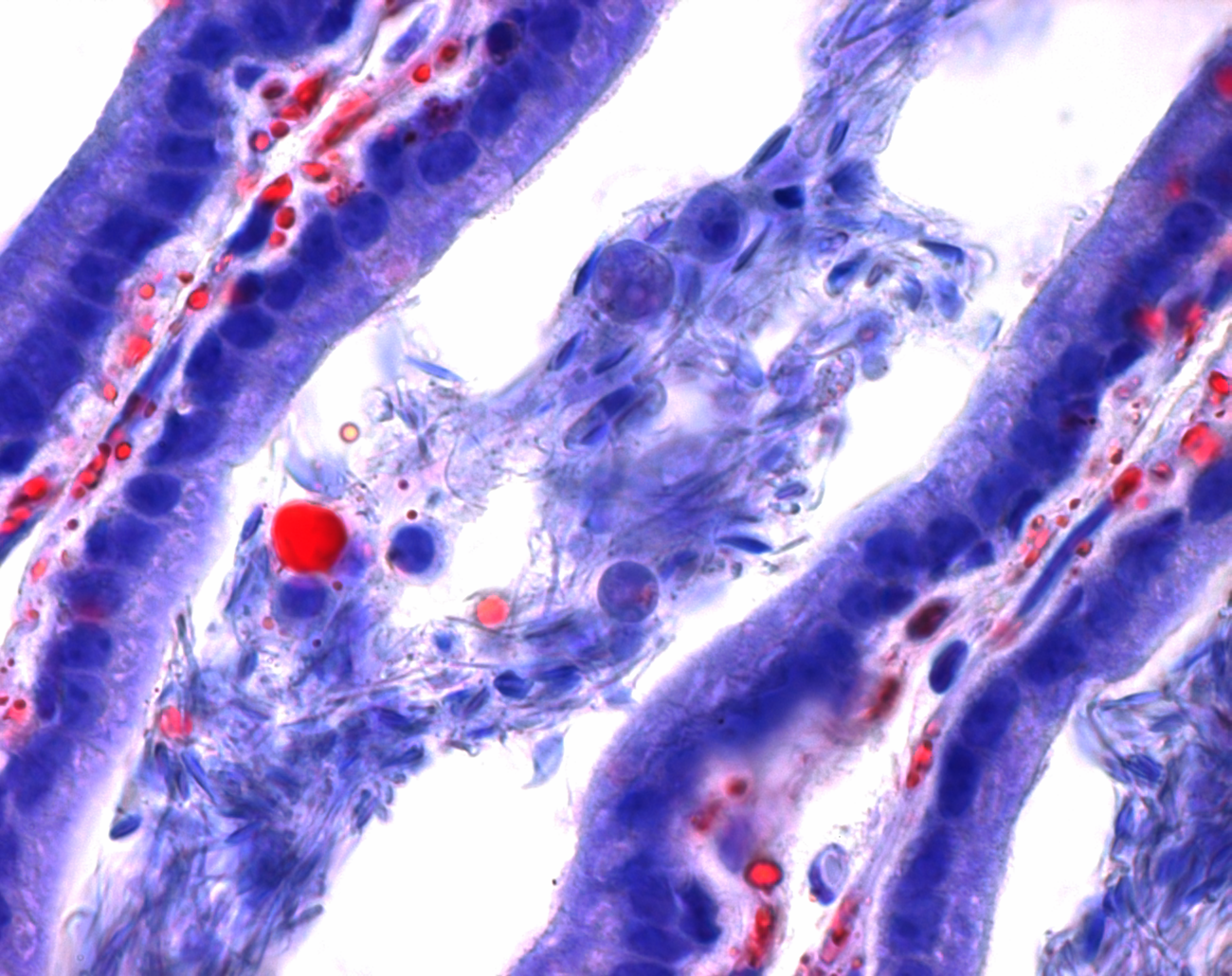
***lxrα;β-/-* 4 months of age, high-cholesterol diet.**

**
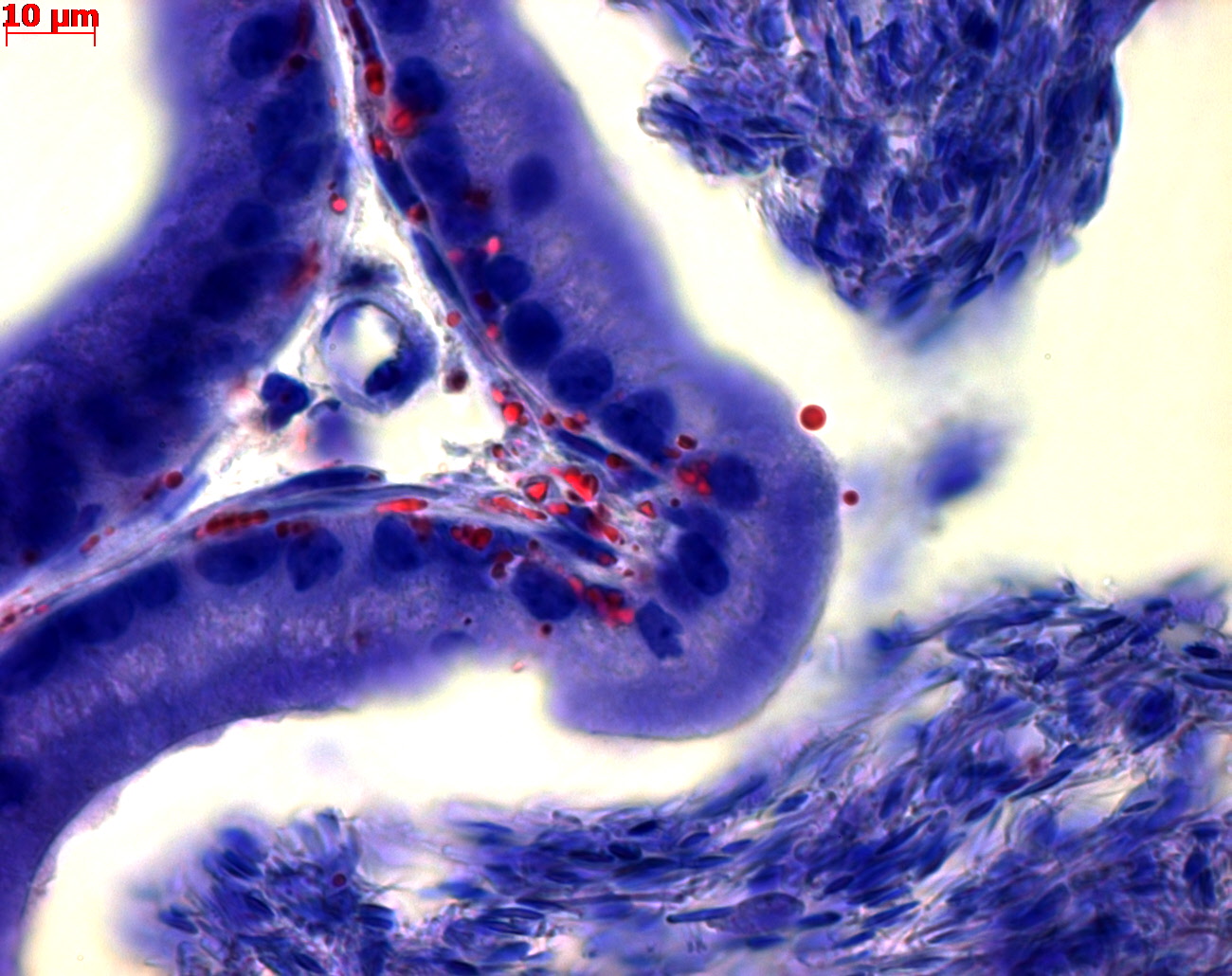
**

**Figure S1**

Supplement: Figure S1 — Neutral lipid accumulation in peritubular smooth muscle cells from LXR-deficient 4-month-old animals fed the control diet (upper panel) or with the high-cholesterol diet for 1 month (lower panel). Oil red O staining of 7 µm cryosections revealing neutral lipids in red (triglycerides and cholesteryl esters). Highly lipid-loaded smooth muscle cells as well as protruding cells are visible in the lower panel. Scale bars represent 10 µm. (DOC) [file pone.0026966.s001.doc]
