# Supplementary material for: Dietary Cholesterol-Induced Post-Testicular Infertility
Source: PLoS One. 2011 Nov 2;6(11):e26966. doi: 10.1371/journal.pone.0026966 (PMC3206870; doi:10.1371/journal.pone.0026966)
Supplement: Figure S2 — Plasma LDL increases in high-cholesterol diet treated mice and in ageing lxrα;β−/− mice. Plasma cholesterol, triglycerides, HDL and LDL concentrations were measured (as described in Text S1) in (A) wt and lxrα;β−/− 4-month old mice fed the control or the high-cholesterol diet and (B) wt and lxrα;β−/− ageing mice at 4, 6 and 8 months of age. Histograms are expressed as mean ± SEM, n = 3. *p<0.05; **p<0.01. (DOC) [file pone.0026966.s002.doc]

**A B**


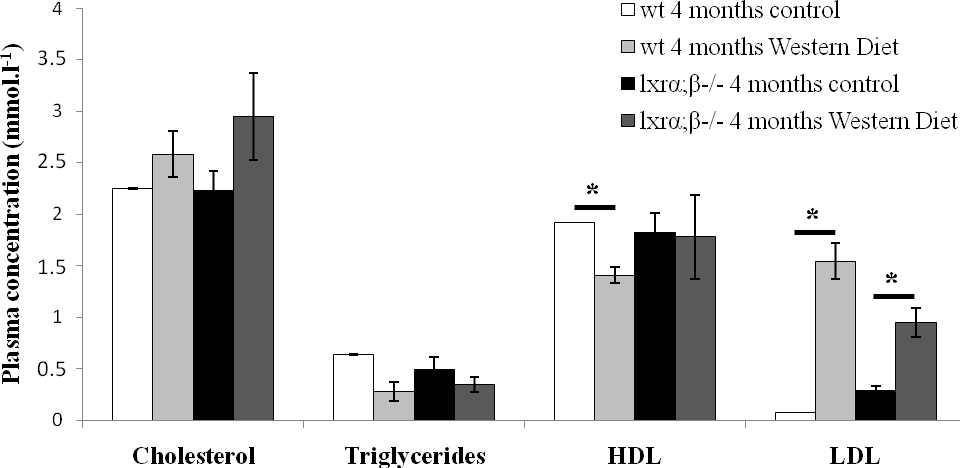


***wt* 4 months control**

***lxrα;β^-/-^* 4 months control**

***wt* 4 months HCD**

***lxrα;β^-/-^* 4 months HCD**


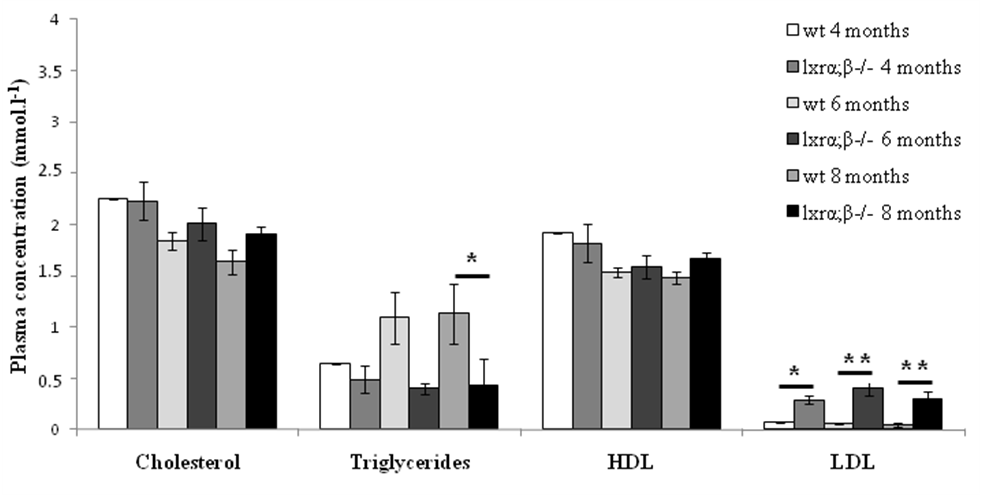


***wt* 4 months**

***wt 6* months**

***lxrα;β^-/-^* 4 months**

***lxrα;β^-/-^* 6 months**

***wt 8* months**

***lxrα;β^-/-^* 8 months**

**Figure S2**
