# Supplementary material for: Dietary Cholesterol-Induced Post-Testicular Infertility
Source: PLoS One. 2011 Nov 2;6(11):e26966. doi: 10.1371/journal.pone.0026966 (PMC3206870; doi:10.1371/journal.pone.0026966)
Supplement: Figure S3 — Modification of caput epididymidal segment 2 SMC markers in high-cholesterol diet-fed 4-month-old lxrα;β−/− animals. Caveolin-1 and smα-actin immunoperoxidase staining are decreased (arrowheads in the bottom panel microphotographs) in segment 2 (S2) of the caput epididymidis from lxrα;β−/− mice at 4 months of age after four weeks of high-cholesterol diet. Inset represents negative control, scale bars represent 10 µm, n = 3. (DOC) [file pone.0026966.s003.doc]

**Control**

**High-cholesterol-diet**

**caveolin-1**

**smα-actin**

**Control**

**High-cholesterol-diet**

**Figure S3**
