# Supplementary material for: Dietary Cholesterol-Induced Post-Testicular Infertility
Source: PLoS One. 2011 Nov 2;6(11):e26966. doi: 10.1371/journal.pone.0026966 (PMC3206870; doi:10.1371/journal.pone.0026966)
Supplement: Text S1 — (DOCX) [file pone.0026966.s004.docx]

**Supporting Information text.S1**

**Protocols:**

**Cholesterol, triglyceride and lipoprotein plasma concentrations (Fig. S2).**

Blood was kept on ice in heparin-coated tubes, centrifuged 15 min at 1500g at 4°C and then plasma was recovered and kept at -80°C until use. Analyses were performed on an automated clinical chemistry analyzer (Hitachi Modular; Roche Diagnostic) based on enzymatic colorimetric assays.
